# A Pilot Study Showing Acute Inhibitory Effect of GLP‐1 on the Bone Resorption Marker CTX in Humans

**DOI:** 10.1002/jbm4.10209

**Published:** 2019-08-23

**Authors:** Anne Nissen, Simone Marstrand, Kirsa Skov‐Jeppesen, Lasse Bremholm, Mads Hornum, Ulrik B Andersen, Jens Juul Holst, Mette Marie Rosenkilde, Bolette Hartmann

**Affiliations:** ^1^ Department of Biomedical Sciences The Panum Institute, University of Copenhagen Copenhagen Denmark; ^2^ NNF Center for Basic Metabolic Research The Panum Institute, University of Copenhagen Copenhagen Denmark; ^3^ Department of Surgery (Gastroenterology Section) Zealand University Hospital, University of Copenhagen Copenhagen Denmark; ^4^ Department of Nephrology Rigshospitalet Copenhagen Denmark; ^5^ Institute for Clinical Medicine University of Copenhagen Copenhagen Denmark; ^6^ Department of Clinical Physiology and Nuclear Medicine and PET Rigshospitalet (Glostrup Section), University of Copenhagen Copenhagen Denmark

**Keywords:** BONE RESORPTION, BONE REMODELING, GLUCAGON‐LIKE PEPTIDE‐1, CTX, EXENATIDE

## Abstract

Bones have been suggested to be a target for glucagon‐like peptide ‐1 (GLP‐1); however, studies of the effects on human bones so far have given diverging results. We hypothesized that GLP‐1, together with glucagon‐like peptide‐2 and glucose‐dependent insulinotropic polypeptide, plays a role in the gut–bone axis. We examined the acute effect of three GLP‐1 receptor ligands [GLP‐1 (7‐36)amide, GLP‐1 (9‐36)amide, and exenatide] on markers of bone remodeling. Eight healthy, normal‐weight participants, with a mean age of 24.3 years, were studied for 4 days in a double‐blinded, randomized clinical trial. Blood was collected before and after s.c. injection of GLP‐1 (7‐36)amide (1.5 nmol/kg), GLP‐1 (9‐36)amide (1.5 nmol/kg), exenatide (2.4 nmol/subject), or saline. Plasma was analyzed for bone markers and for osteoprotegerin (OPG), PTH, and IGF‐1 levels. All ligands were tested in vitro for their cAMP‐inducing activity on the human GLP‐1 receptor. GLP‐1 (7‐36)amide decreased CTX‐levels, compared with placebo (area under the curve [AUC] ±SD 0 to 120 min = –2143 ± 1294 % × min versus –883 ± 1557 % × min; *p* < 0.05). No difference was observed between placebo and GLP‐1 (9‐36)amide, or between placebo and exenatide, although exenatide had a similar potency as GLP‐1 (7‐36)amide for cAMP formation in vitro (EC_50_ of 0.093 and 0.054 nmol/L). However, exenatide reached maximum plasma concentration at 90 min versus 15 min for GLP‐1 (7‐36)amide, and plasma CTX was significantly decreased during the second hour of the study after exenatide injections compared with placebo (AUC ±SD –463.1 ± 218 % × min and –136 ± 91 % × min; *p* < 0.05). There was no effect of the injections on bone formation markers (P1NP and osteocalcin) or on OPG, PTH and IGF‐1 levels. In conclusion, we show that GLP‐1 receptor agonists, but not the primary metabolite GLP‐1 (9‐36)amide, decrease bone resorption, and suggest that GLP‐1 may be part of the gut–bone axis. © 2019 The Authors. *JBMR Plus* is published by Wiley Periodicals, Inc. on behalf of the American Society for Bone and Mineral Research.

## Introduction

Glucagon‐like peptide‐1 (GLP‐1) is a peptide hormone secreted from enteroendocrine L‐cells upon meal ingestion.^1^ Active GLP‐1, GLP‐1 (7‐36)amide, has an apparent plasma half‐life of approximately 2 min, as it is rapidly degraded to GLP‐1 (9‐36)amide by the enzyme, dipeptidyl peptidase‐4 (DPP‐4).^2^

GLP‐1 is primarily known as an insulinotropic hormone, which together with glucose‐dependent insulinotropic polypeptide (GIP), is responsible for the incretin effect, ie, the markedly increased insulin secretion after oral as opposed to i.v.‐administered glucose.^3^ The effect of GLP‐1 on insulin secretion is being exploited in the treatment of type 2 diabetes mellitus, where GLP‐1 receptor agonists (GLP‐1RAs) are widely used.

The metabolite, GLP‐1 (9‐36)amide, has little effect on insulin secretion or glucose metabolism in humans.^4^, ^5^, ^6^ In fact, GLP‐1 (9‐36)amide is a weak antagonist of the GLP‐1 receptor.^7^ However, rodent studies have suggested protective cardiovascular effects of GLP‐1 (9‐36)amide, perhaps independent of GLP‐1 receptor signaling.^8^

Extrapancreatic effects of GLP‐1 (7‐36)amide include the inhibition of appetite, food intake, and gastrointestinal motility^3^; nevertheless, it has beneficial effects on the cardiovascular system.^9^ Finally, recent studies have suggested an effect of GLP‐1 on bone remodeling.^10^, ^11^

Bone remodeling shows a diurnal variation, in particular, a decrease in bone resorption is observed postprandially, which can be eliminated by fasting.^12^, ^13^ Clowes and colleagues showed that the postprandial decrease in bone resorption also can be eliminated by the administration of a somatostatin analogue before meal ingestion.^14^ Another effect of somatostatin is to powerfully inhibit the secretion of gut hormones, GLP‐1, glucagon‐like peptide‐2 (GLP‐2), and GIP. Both GIP and GLP‐2 acutely decrease CTX plasma levels^15^, ^16^; therefore, we hypothesized that the highly related hormone GLP‐1 might have a similar effect.

A study in ovariectomized mice showed that GLP‐1 administration was associated with an increase in BMD, decreases in RANKL expression, and increases in the bone formation markers, osteocalcin (OC), and P1NP secretion.^17^, ^18^ The effect of GLP‐1 on bones in rats and mice has been suggested (1) to involve increased calcitonin secretion,^19^ or (2) to represent a direct effect on osteocytes leading to a decrease in sclerostin formation.^20^

GLP‐1 receptors have been observed on premature human osteoblasts, but not on mature human osteoblasts.^21^ In mice, GLP‐1 receptors have been observed on both premature and mature osteoblasts.^22^ Nuche‐Berenguer and colleagues found that GLP‐1 binding to the murine osteoblast cell line, MC3T3‐E1, increases glycosylphosphatidylinositol‐/inositolphosphoglycan‐associated activity, but not cAMP levels, as normally seen after activation of the single GLP‐1 receptor.^23^ Yet, in other studies, GLP‐1 receptors could not be demonstrated in murine osteoblasts.^24^, ^25^

The long‐acting GLP‐1RA, liraglutide, has been reported to promote the differentiation of both human and rat bone marrow stromal cells into osteoblasts and to inhibit adipocytogenesis.^17^

In vitro studies show that GLP‐1 may increase the number of osteoclasts, but that their resorptive area decreases, resulting in no effect on the resorption rate.^22^

Diverging effects of GLP‐1 have been found in humans. Liraglutide treatment was associated with a reduced risk of fractures, whereas exenatide was associated with an increased risk of fractures as compared with placebo or other antidiabetic drugs.^11^ However, in a population‐based cohort study, neither liraglutide nor exenatide influenced fracture risk compared with other oral antidiabetic drugs.^26^ Similar results were found in a meta‐analysis by Mabilleau and colleagues.^24^ In a study of weight‐loss maintenance in a group of nondiabetic, obese women, liraglutide prevented weight‐loss‐associated bone loss, and increased the bone formation marker, P1NP, as compared with a control group.^10^ In a study of type 1 diabetics, the acute effect of GLP‐1 on bone remodeling under hypoglycemia was examined, and showed no effect on CTX, P1NP, or PTH.^27^

To elucidate the possible acute effect of GLP‐1RA on bone remodeling, we examined the acute effect of s.c. injections of intact GLP‐1 (7‐36)amide, as well as the metabolite GLP‐1 (9‐36)amide and the degradation resistant GLP‐1RA, exenatide, on bone remodeling.

## Subjects and Methods

### Study population

The study population consisted of eight volunteers (five male), with a mean age of 24.3 years (range 19 to 27, SD 2.7) and a mean BMI of 22.6 (SD 2.1) kg/m^2^. All participants were normotensive and routine blood chemistry analyses showed normal values of creatinine, Na^+^, K^+^, CRP, leucocytes, alanine transaminase, aspartate aminotransferase, alkaline phosphatase, amylase, hemoglobin, glucose, HbA1c, and cholesterol. Data regarding the circulatory effects of GLP‐1 (7‐36)amide, GLP‐1(9‐36)amide, and exenatide, particularly with respect to mesenteric blood flow, were recently published.^28^

### Procedure

The study was conducted as a double‐blinded randomized clinical trial. GLP‐1 (7‐36)amide, GLP‐1 (9‐36)amide, exenatide (exendin‐4), and saline were injected on 4 different study days under identical circumstances. Synthetic human GLP‐1 (9‐36)amide (96% pure; Bachem, Bubendorf, Germany) as well as synthetic human GLP‐1 (7‐36)amide (99% pure; Bachem) were both dissolved in saline with 2% human serum albumin (CSL Behring GmbH, Marburg, Germany), sterile filtered, and dispensed into glass ampules that were afterwards tested for sterility and pyrogenes. The purity and structure of the peptides were examined by sequence, HPLC, and mass analysis. Exenatide (Byetta, AstraZeneca Pharmaceuticals, Cambridge, UK; exendin‐4) and isotonic saline are commercially available and were used without modification.

The participants arrived after an overnight fast; the female participants underwent a urine hCG‐test (which was negative in all cases). The participants were placed in the supine position and an i.v. access was established. Following a resting period (15 to 20 min), subjects received on the 4 study days and in random order: s.c. injections (1 mL) of isotonic saline, 2.4 nmol exenatide, 1.5 nmol/kg synthetic GLP‐1 (9‐36)amide, or 1.5 nmol/kg synthetic GLP‐1 (7‐36)amide.

Venous blood samples were collected in chilled EDTA tubes at time points –15, 0, 15, 30, 45, 60, 90, and 120 min, and centrifuged at 3600 rpm for 10 min at 4°C. Next, plasma was transferred to ice‐chilled tubes (Minisorb, NUNC, Roskilde, Denmark) and stored at –20**°**C until time of analysis. Serum was stored in cryotubes at –80**°**C.

### Blood sample analysis

Radioimmunoassays were used for measuring plasma concentrations of both GLP‐1 (total and intact) and exenatide after ethanol extraction, as described previously.^28^ A glucose analyzer (YellowSprings Instrument, YSI Inc., Yellow Springs, OH, USA) was used for measuring blood glucose by the glucose oxidase method.

Plasma insulin and C‐peptide were measured using a Cobas 8000, e602 module (Roche Diagnostics GmbH, Mannheim, Germany), using the following Cobas products: insulin reagents, insulin calibrator, C‐peptide reagents, and C‐peptide calibrator. The Cobas module uses a sandwich electro‐chemiluminescense immunoassay.

Plasma samples were analyzed for OC, PTH, IGF‐1, CTX, and P1NP on an ids‐iSYS Multi‐ Discipline Automated System (Immunodiagnostic Systems, Copenhagen, Denmark) by the automated chemiluminescence immunoassay method. The method is FDA‐cleared and CE marked. Osteoprotegerin (OPG) was measured using a commercially available sandwich ELISA kit (Osteoprotegerin ELISA kit, rev. no. 150806, Biomedica Medizinprodukte GmbH, Vienna, Austria). The ELISA uses specific monoclonal antibodies towards human OPG.

### cAMP measurements in vitro

COS‐7 cells were cultured at 10% CO_2_ and 37°C in Dulbecco's modified Eagles medium 1885 supplemented with 10% FBS, 2 mmol/L glutamine, 180 units/mL penicillin, and 45 g/mL streptomycin. Transient transfection of the human GLP‐1 receptor was performed using the calcium phosphate precipitation method.^29^ The transfected COS‐7 cells were seeded in 96‐well plates one day after transfection (35,000 cells/well) and the experiments carried out the following day. In short, the cells were washed twice with HEPES‐buffered saline (HBS) buffer and incubated with HBS and 1 mmol/L 3‐isobutyl‐1‐methylxanthine (IBMX) for 30 min at 37°C.^30^ The three ligands were added in increasing concentrations and incubated for 30 min at 37°C. The HitHunter cAMP XS assay (Eurofins DiscoverX Corp, Fremont, CA, USA) was carried out according to the manufacturer's instructions. In vitro pharmacological analyses were carried out with the GraphPad Prism (GraphPad Software, La Jolla, CA, USA). Sigmoid curves were fitted logistically with a Hill slope of 1.0.

### Statistics

Based on previous studies,^16^, ^31^, ^32^ we calculated that a minimum of eight participants would be necessary to detect a difference of 20% in CTX with a power of 85%, two‐sided 5% significance level, and a SD of 13%. The results are expressed as percentage of fasting level, being the mean value of measurements at time point –15 and 0. Areas under the curve (AUC) have been calculated with *y* = 100% as baseline. Differences in AUCs and plasma levels of the hormones were examined by one‐way ANOVA for repeated measurements, followed by Dunnett's multiple comparison test. Differences resulting in a *p* value <0.05 were considered significant. Calculations and graphs were all made in GraphPad Prism 5 (GraphPad Software).

### Ethics

The study was approved by the Regional Committee on Biomedical Research (SJ‐339). The study was performed in concordance with the Helsinki II Declaration, as well as the Danish Data Protection Agency. All participants gave oral and written consent to participate. The study has been registered at clinicaltrials.gov, Protocol Registration number NCT01988545.

## Results

Plasma concentrations of GLP‐1 (7‐36)amide, GLP‐1 (9‐36)amide, exenatide, glucose, insulin, and C‐peptide were presented previously.^28^ GLP‐1 (7‐36)amide injection led to a rise in total plasma concentration reaching a maximum mean value of 316 pmol/L after 15 min, thereafter it declined, but was still elevated after 120 min. GLP‐1 (9‐36)amide reached a mean plasma concentration of 290 pmol/L at 30 min, and decreased to near baseline levels during the study. The exenatide injection resulted in a plateau concentration of approximately 300 pmol/L from 45 to 120 min, with a maximal mean plasma concentration of 313 pmol/L at 90 min.

**Figure 1:**
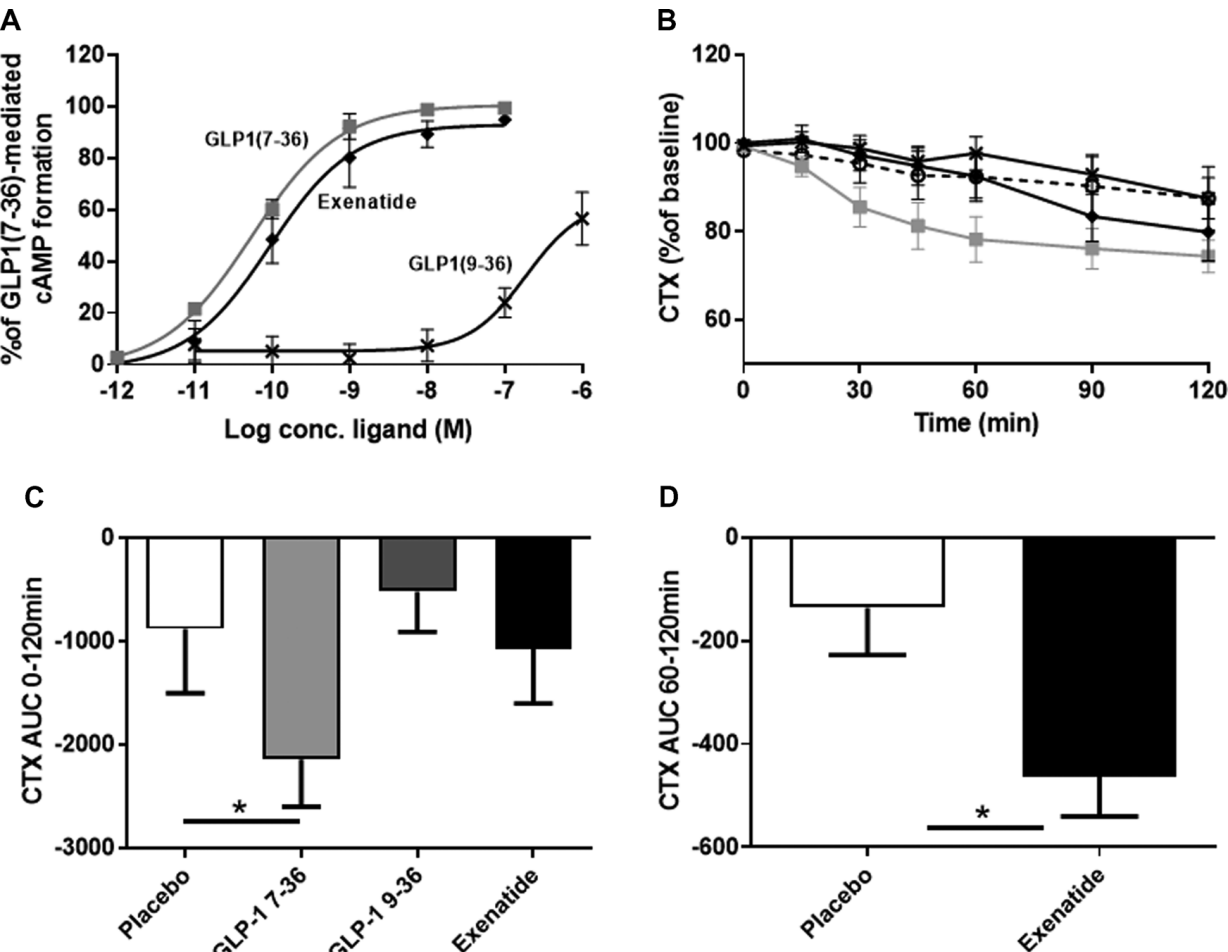
GLP‐1 effect on in vitro signaling and on CTX in humans. (*A*) cAMP formation in transiently transfected COS‐7 cells expressing the human GLP‐1R. (*B*) Mean CTX levels ±SEM shown as percent of basal level (calculated as mean of –10 and 0 min). (*C*) Mean AUC (area under the curve) time 0 to 120 min ±SEM, *p < 0.05. (*D*) Mean AUC exenatide and placebo, time 60 to 120 min ±SEM, *p < 0.05. Grey square = GLP‐1 (7‐36)amide*;* black cross *=* GLP‐1 (9‐36)amide; black diamond = exenatide; open circle, dashed line = placebo.

In vitro, all three GLP‐1 ligands acted as agonists of the GLP‐1 receptor. GLP‐1 (7‐36)amide and exenatide acted as full agonists for cAMP formation with EC_50_ of 0.093 and 0.054 nmol/L, respectively, whereas GLP‐1(9‐36)amide acted as a low potency partial agonist with an estimated EC_50_ of 188 nmol/L (Fig. 1 *A*), as no Emax was reached even at 1 µmol/L GLP‐1(9‐36)amide.

Injection of the two strong agonists resulted in a decrease in the bone resorption marker CTX (Fig. 1 *B*). The GLP‐1 (7‐36)amide injections resulted in a gradual decrease of CTX levels reaching 74.4% (SD ±10.4%) of the fasting level at 120 min, with a significant difference in the AUC_0‐120min_ (±SD) between GLP‐1 (7‐36)amide (–2143 ± 1294 % × min) and placebo (–883 ± 1757% × min; Fig. 1 *C*). Exenatide injections resulted in plasma levels of CTX of 79.8% (SD ± 18.6%) at 120 min. When measured over the entire period, there was no significant difference in AUC between exenatide compared with the placebo day. However, calculating the AUC during the second hour of the study (60 to 120 min; ie, when plasma exenatide levels reached maximum) revealed a significantly larger AUC (±SD) in the exenatide group compared with placebo (–463 ± 218% × min and –136 ± 258% × min respectively; *p* < 0.05; Fig. 1 *D*). The partial agonist, GLP‐1 (9‐36)amide, did not result in any significant changes in CTX over the entire period in comparison to placebo as the CTX level reached 87.5% (SD ± 12.4%) of fasting level at 120 min for GLP‐1 (9‐36)amide injections compared with 87.5 % (SD ± 20.4%) upon saline injections.

Absolute baseline values of CTX were 0.63 ± 0.33, 0.6 ± 0.25, 0.64 ± 0.33, and 0.59 ± 0.31 ng/mL on the study days with GLP‐1 (7‐36)amide, GLP‐1 (9‐36)amide, exenatide, and placebo, respectively (one way ANOVA *p* = 0.99).

**Figure 2:**
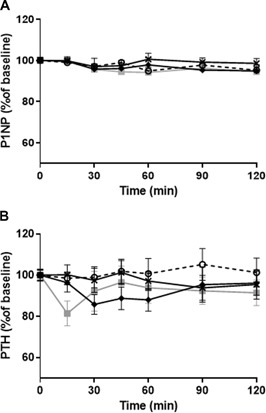
GLP‐1 effect on P1NP and PTH. (*A*) Mean P1NP levels ±SEM shown as percent of basal level (calculated as mean of –10 and 0). (*B*) Mean PTH levels ±SEM shown as percent of basal level (calculated as mean of –10 and 0 min). Grey square = GLP‐1 (7‐36)amide; black cross = GLP‐1 (9‐36)amide;black diamond = exenatide; open circle, dashed line = placebo.

The bone formation marker P1NP showed very little variation from the baseline level during the study (Fig. 2 *A*), and none of the injections led to significant changes compared with placebo.

PTH did show some variation from baseline as apparent from Fig. 2 *B*; however, there were no significant differences between any of the hormones and placebo. Moreover, we found no effect of any of the injections on OC, IGF‐1, and OPG (data not shown).

## Discussion

The present study found an acute decrease in CTX after injection of GLP‐1 (7‐36)amide, whereas the metabolite GLP‐1(9‐36)amide had no impact. In addition, exenatide injections resulted in a decrease in CTX, but only during the last half of the experiment. However, the s.c. injections of exenatide resulted in a much slower increase in plasma concentrations as compared with GLP‐1 (7‐36)amide with peak concentrations at 90 min compared with 15 min for GLP‐1(7‐36)amide. This slower increase is probably because of the much lower dose and the extended half‐life of exenatide compared with GLP‐1 (7‐36)amide. Thus, the decrease in CTX after exenatide closely followed the plasma profile and was significantly different from placebo only in the last half of the experiments (60 to 120 min).

The effect of GLP‐1 (7‐36)amide on bone resorption found in this study contrasts with the results of a study by Christensen and colleagues, where GLP‐1 did not have any acute effect on bone resorption.^27^ However, that study was done in individuals with type 1 diabetes under hypoglycemic conditions and using lower doses, which may explain the apparent differences.

The findings in the present study are also in opposition to a previous study by Henriksen and colleagues^16^ that reported no acute effect of GLP‐1. In that study they actually found similar decreases in CTX after GLP‐1, but missed significance, because of lack of a control group.

We also examined the GLP‐1 metabolite, GLP‐1 (9‐36)amide, and found no effect of this on any of the markers of bone remodeling. This is in concordance with the very low potency observed for GLP‐1 (9‐36)amide regarding GLP‐1 receptor activation and the plasma concentrations reached (approximately 300 pmol/L), which were probably too low for sufficient activation of the GLP‐1 receptor. Previous studies have likewise described low potency, and partial agonistic properties of GLP‐1 (9‐36)amide.^33^ Indeed, both in vitro and in vivo, it has antagonistic properties.^7^ The negative results are, nevertheless, of interest in view of the remarkable reported cardiovascular effects of this metabolite.^8^

Previous studies of postprandial bone remodeling show that oral glucose (eg, in the form of an oral glucose tolerance test) can lead to a decrease in bone resorption as measured by CTX levels to as low as 50% of the fasting level (baseline level).^14^, ^34^ Here a decrease to 74% of the baseline level was reached after injections of GLP‐1 (7‐36)amide, which indicates that GLP‐1 is not responsible for the entire postprandial inhibition of bone resorption. This leaves room for an effect of other gut hormones, such as GIP and GLP‐2, which both have been suggested as major players in the gut–bone axis.^15^, ^16^

A strength of the present study is that it was done in healthy young people, with normal glucose tolerance, eliminating skewed results because of, ie, lowered GLP‐1 sensitivity (as seen in patients with type 2 diabetes).^35^ Another strength is that the bone remodeling process was examined thoroughly by analyzing a range of different markers and hormones (CTX, OPG, PTH, OC, P1NP, and IGF‐1). The main limitation of the study is the relatively low number of participants (*n* = 8). Nevertheless, the group was large enough to reveal significant effects of the two agonists, whereas there was no indication whatsoever of an effect of the GLP‐1 metabolite. Furthermore, it might have been beneficial to increase the duration of the observation period because the CTX levels did not return (increase) to fasting levels following the GLP‐1‐mediated decrease in bone resorption. However, in previous studies of longer duration the nadir of CTX was reached at 120 min after oral glucose intake.^14^, ^34^ Another limitation is the possible effect of GLP‐1 on the kidneys. In theory, the observed effect on CTX levels could be caused by increased clearance induced by GLP‐1 of collagen degradation fragments, which are normally eliminated by glomerular filtration. However, GLP‐1 does not seem to alter renal artery flow^28^ or renal plasma flow and glomerular filtration rate in humans.^36^

In conclusion, the present study shows an inhibitory effect of the two GLP‐1R agonists, GLP‐1 (7‐36)amide and exenatide, on bone resorption in humans. GLP‐1 may therefore contribute to the regulation of bone turnover as part of the gut–bone axis together with the more established gut hormone mediators, GIP and GLP‐2.

## Disclosures

The authors declare that they have nothing to disclose and no conflicts of interest associated with this manuscript.

## References

[1] Orskov C , Holst JJ , Nielsen OV . Effect of truncated glucagon‐like peptide‐1 [proglucagon‐(78‐107) amide] on endocrine secretion from pig pancreas, antrum, and nonantral stomach. Endocrinology. 1988;123(4):2009–13. 2901341 10.1210/endo-123-4-2009

[2] Deacon CF , Johnsen AH , Holst JJ . Degradation of glucagon‐like peptide‐1 by human plasma in vitro yields an N‐terminally truncated peptide that is a major endogenous metabolite in vivo. J Clin Endocrinol Metab. 1995;80(3):952–7. 7883856 10.1210/jcem.80.3.7883856

[3] Holst JJ . The physiology of glucagon‐like peptide 1. Physiol Rev. 2007;87(4):1409–39. 17928588 10.1152/physrev.00034.2006

[4] Sathananthan M , Farrugia LP , Miles JM , et al. Direct effects of exendin‐(9,39) and GLP‐1‐(9,36)amide on insulin action, beta‐cell function, and glucose metabolism in nondiabetic subjects. Diabetes. 2013;62(8):2752–6. 23545708 10.2337/db13-0140 PMC3717878

[5] Zander M , Madsbad S , Deacon CF , Holst JJ . The metabolite generated by dipeptidyl‐peptidase 4 metabolism of glucagon‐like peptide‐1 has no influence on plasma glucose levels in patients with type 2 diabetes. Diabetologia. 2006;49(2):369–74. 16385384 10.1007/s00125-005-0098-y

[6] Meier JJ , Gethmann A , Nauck MA , et al. The glucagon‐like peptide‐1 metabolite GLP‐1‐(9‐36) amide reduces postprandial glycemia independently of gastric emptying and insulin secretion in humans. Am J Physiol Endocrinol Metab. 2006;290(6):E1118–23. 16403774 10.1152/ajpendo.00576.2005

[7] Knudsen LB , Pridal L . Glucagon‐like peptide‐1‐(9‐36) amide is a major metabolite of glucagon‐like peptide‐1‐(7‐36) amide after in vivo administration to dogs, and it acts as an antagonist on the pancreatic receptor. Eur J Pharmacol. 1996;318(2–3):429–35. 9016935 10.1016/s0014-2999(96)00795-9

[8] Ban K , Noyan‐Ashraf MH , Hoefer J , Bolz SS , Drucker DJ , Husain M . Cardioprotective and vasodilatory actions of glucagon‐like peptide 1 receptor are mediated through both glucagon‐like peptide 1 receptor‐dependent and ‐independent pathways. Circulation. 2008;117(18):2340–50. 18427132 10.1161/CIRCULATIONAHA.107.739938

[9] Nauck MA , Meier JJ , Cavender MA , Abd El AM , Drucker DJ . Cardiovascular actions and clinical outcomes with glucagon‐like peptide‐1 receptor agonists and dipeptidyl peptidase‐4 inhibitors. Circulation. 2017;136(9):849–70. 28847797 10.1161/CIRCULATIONAHA.117.028136

[10] Iepsen EW , Lundgren JR , Hartmann B , et al. GLP‐1 Receptor agonist treatment increases bone formation and prevents bone loss in weight‐reduced obese women. J Clin Endocrinol Metab. 2015;100(8):2909–17. 26043228 10.1210/jc.2015-1176

[11] Su B , Sheng H , Zhang M , et al. Risk of bone fractures associated with glucagon‐like peptide‐1 receptor agonists' treatment: a meta‐analysis of randomized controlled trials. Endocrine. 2015;48(1):107–15. 25074632 10.1007/s12020-014-0361-4

[12] Christgau S . Circadian variation in serum CrossLaps concentration is reduced in fasting individuals. Clin Chem. 2000;46(3):431. 10702538

[13] Qvist P , Christgau S , Pedersen BJ , Schlemmer A , Christiansen C . Circadian variation in the serum concentration of C‐terminal telopeptide of type I collagen (serum CTx): effects of gender, age, menopausal status, posture, daylight, serum cortisol, and fasting. Bone. 2002;31(1):57–61. 12110413 10.1016/s8756-3282(02)00791-3

[14] Clowes JA , Allen HC , Prentis DM , Eastell R , Blumsohn A . Octreotide abolishes the acute decrease in bone turnover in response to oral glucose. J Clin Endocrinol Metab. 2003;88(10):4867–73. 14557467 10.1210/jc.2002-021447

[15] Nissen A , Christensen M , Knop FK , Vilsboll T , Holst JJ , Hartmann B . Glucose‐dependent insulinotropic polypeptide inhibits bone resorption in humans. J Clin Endocrinol Metab. 2014;99(11):E2325–29. 25144635 10.1210/jc.2014-2547

[16] Henriksen DB , Alexandersen P , Bjarnason NH , et al. Role of gastrointestinal hormones in postprandial reduction of bone resorption. J Bone Miner Res. 2003;18(12):2180–9. 14672353 10.1359/jbmr.2003.18.12.2180

[17] Lu N , Sun H , Yu J , et al. Glucagon‐like peptide‐1 receptor agonist liraglutide has anabolic bone effects in ovariectomized rats without diabetes. PLoS One. 2015;10(7):e0132744. 26177280 10.1371/journal.pone.0132744 PMC4503456

[18] Ma X , Meng J , Jia M , et al. Exendin‐4, a glucagon‐like peptide‐1 receptor agonist, prevents osteopenia by promoting bone formation and suppressing bone resorption in aged ovariectomized rats. J Bone Miner Res. 2013;28(7):1641–52. 23427056 10.1002/jbmr.1898

[19] Yamada C , Yamada Y , Tsukiyama K , et al. The murine glucagon‐like peptide‐1 receptor is essential for control of bone resorption. Endocrinology. 2008;149(2):574–9. 18039776 10.1210/en.2007-1292

[20] Kim JY , Lee SK , Jo KJ , et al. Exendin‐4 increases bone mineral density in type 2 diabetic OLETF rats potentially through the down‐regulation of SOST/sclerostin in osteocytes. Life Sci. 2013;92(10):533–40. 23357248 10.1016/j.lfs.2013.01.001

[21] Pacheco‐Pantoja EL , Ranganath LR , Gallagher JA , Wilson PJ , Fraser WD . Receptors and effects of gut hormones in three osteoblastic cell lines. BMC Physiol. 2011;11:12. 21801348 10.1186/1472-6793-11-12 PMC3162581

[22] Pereira M , Jeyabalan J , Jorgensen CS , et al. Chronic administration of glucagon‐like peptide‐1 receptor agonists improves trabecular bone mass and architecture in ovariectomised mice. Bone. 2015;81:459–67. 26314515 10.1016/j.bone.2015.08.006

[23] Nuche‐Berenguer B , Portal‐Nunez S , Moreno P , et al. Presence of a functional receptor for GLP‐1 in osteoblastic cells, independent of the cAMP‐linked GLP‐1 receptor. J Cell Physiol. 2010;225(2):585–92. 20506394 10.1002/jcp.22243

[24] Mabilleau G , Mieczkowska A , Chappard D . Use of glucagon‐like peptide‐1 receptor agonists and bone fractures: a meta‐analysis of randomized clinical trials. J Diabetes. 2014;6(3):260–6. 24164867 10.1111/1753-0407.12102

[25] Meng J , Ma X , Wang N , et al. Activation of GLP‐1 receptor promotes bone marrow stromal cell osteogenic differentiation through beta‐catenin. Stem Cell Rep. 2016;6(4):579–91. 10.1016/j.stemcr.2016.02.002 PMC4834036 26947974

[26] Driessen JH , van Onzenoort HA , Starup‐Linde J , et al. Use of glucagon‐like‐peptide 1 receptor agonists and risk of fracture as compared to use of other anti‐hyperglycemic drugs. Calcif Tissue Int. 2015;97(5):506–15. 26184119 10.1007/s00223-015-0037-y PMC4598352

[27] Christensen MB , Lund A , Calanna S , et al. Glucose‐dependent insulinotropic polypeptide (gip) inhibits bone resorption independently of insulin and glycemia. J Clin Endocrinol Metab. 2018;103(1):288–94. 29099978 10.1210/jc.2017-01949

[28] Bremholm L , Andersen UB , Hornum M , et al. Acute effects of glucagon‐like peptide‐1, GLP‐19‐36 amide, and exenatide on mesenteric blood flow, cardiovascular parameters, and biomarkers in healthy volunteers. Physiol Rep. 2017;5(4). 10.14814/phy2.13102 PMC5328764 28235974

[29] Hansen LS , Sparre‐Ulrich AH , Christensen M , et al. N‐terminally and C‐terminally truncated forms of glucose‐dependent insulinotropic polypeptide are high‐affinity competitive antagonists of the human GIP receptor. Br J Pharmacol. 2016;173(5):826–38. 26572091 10.1111/bph.13384 PMC4761099

[30] Hassing HA , Fares S , Larsen O , et al. Biased signaling of lipids and allosteric actions of synthetic molecules for GPR119. Biochem Pharmacol. 2016;119: 66–75. 27569424 10.1016/j.bcp.2016.08.018

[31] Askov‐Hansen C , Jeppesen PB , Lund P , Hartmann B , Holst JJ , Henriksen DB . Effect of glucagon‐like peptide‐2 exposure on bone resorption: effectiveness of high concentration versus prolonged exposure. Regul Pept. 2013;181:4–8. 23261963 10.1016/j.regpep.2012.11.002

[32] Gottschalck IB , Jeppesen PB , Holst JJ , Henriksen DB . Reduction in bone resorption by exogenous glucagon‐like peptide‐2 administration requires an intact gastrointestinal tract. Scand J Gastroenterol. 2008;43(8):929–37. 19086164 10.1080/00365520801965381

[33] Montrose‐Rafizadeh C , Yang H , Rodgers BD , Beday A , Pritchette LA , Eng J . High potency antagonists of the pancreatic glucagon‐like peptide‐1 receptor. J Biol Chem. 1997;272(34):21201–6. 9261127 10.1074/jbc.272.34.21201

[34] Westberg‐Rasmussen S , Starup‐Linde J , Hermansen K , et al. Differential impact of glucose administered intravenously or orally on bone turnover markers in healthy male subjects. Bone. 2017;97: 261–6. 28126633 10.1016/j.bone.2017.01.027

[35] Hojberg PV , Vilsboll T , Rabol R , et al. Four weeks of near‐normalisation of blood glucose improves the insulin response to glucagon‐like peptide‐1 and glucose‐dependent insulinotropic polypeptide in patients with type 2 diabetes. Diabetologia. 2009;52(2):199–207. 19037628 10.1007/s00125-008-1195-5

[36] Asmar A , Simonsen L , Asmar M , et al. Renal extraction and acute effects of glucagon‐like peptide‐1 on central and renal hemodynamics in healthy men. Am J Physiol Endocrinol Metab. 2015;308(8):E641–49. 25670826 10.1152/ajpendo.00429.2014

